# A systemic analysis of monocarboxylate transporters in ovarian cancer and possible therapeutic interventions

**DOI:** 10.1080/19336950.2023.2273008

**Published:** 2023-11-07

**Authors:** Priti Chatterjee, Debaleena Bhowmik, Sib Sankar Roy

**Affiliations:** a Cell Biology and Physiology Division, CSIR‐Indian Institute of Chemical Biology, Kolkata, India; b Academy of Scientific and Innovative Research (AcSIR), Ghaziabad - 201002, India

**Keywords:** Monocarboxylate transporters (MCTs), SLC16A family, ovarian cancer, lactate, differential expression analysis, TCGA (the cancer Genome Atlas)

## Abstract

Monocarboxylate transporters (MCTs) play an immense role in metabolically active solid tumors by regulating concentration-dependent transport of different important monocarboxylates including pyruvate and lactate and are encoded by the SLC16A family of genes. Given the vast array of functions, these transporters play in oncogenesis, our objective was to look into the association of MCT1 (SLC16A1), MCT2 (SLC16A7), MCT3 (SLC16A8), and MCT4 (SLC16A3) with Epithelial ovarian cancer (EOC) pathophysiology by exploiting various publicly available databases and web resources. Few of the *in silico* findings were confirmed via *in vitro* experiments in EOC cell lines, SKOV3 and OAW-42. MCT1 and MCT4 were found to be upregulated at the mRNA level in OC tissues compared to normal. However, only higher level of MCT4 mRNA was found to be associated with poor patient survival. MCT4 was positively correlated with gene families responsible for invasion, migration, and immune modification, proving it to be one of the most important MCTs for therapeutic intervention. We compared the effects of MCT1/2 blocker SR13800 and a broad-spectrum MCT blocker α-Cyano Hydroxy Cinnamic Acid (α-CHCA) and discovered that α-CHCA has a greater effect on diminishing the invasive behavior of the cancer cells than MCT1/2 blocker SR13800. From our study, MCT4 has emerged as a prospective marker for predicting poor patient outcomes and a potential therapeutic target.

## Introduction

Monocarboxylate transporters (MCTs) represent a sub-family of solute carrier proteins (SLCs) which are essentially involved in nutrient and metabolite transport across the plasma membrane of the cells. The SLC16A gene family of MCTs is associated with the transport of monocarboxylates such as lactate and pyruvate and are highly involved in metabolic processes and maintaining pH homeostasis [1]. Out of 14 members of the SLC16A family, only four genes code for MCT proteins that are well-characterized, namely, MCT1 (SLC16A1), MCT2 (SLC16A7), MCT3 (SLC16A8), and MCT4 (SLC16A3). Only these MCT1–4 have the capability to catalyze the proton-linked transport of monocarboxylate entities to maintain cellular homeostasis [1,2].

As the vital components of the central carbon metabolism, the MCTs have gained immense importance over the past few years in cancer-metabolism related research. Cancer cells mostly rely on aerobic glycolysis, where the glucose flux entering the cells is mostly channelized to form lactate and exported out of the cells. The co-transport of lactate with protons helps to maintain the acidic pH, aiding the activation of different metalloproteases needed for tissue invasion by the malignant cells [3]. Different MCTs have different localization, tissue specificity, and substrate affinity and are mostly altered in solid tumor tissues, making them prospective therapeutic targets [4].

Ovarian cancer remains one of the major causes behind gynecological cancer-related deaths worldwide and the Epithelial Ovarian cancer or EOC is the most abundant and lethal form of Ovarian cancer [5,6]. Several factors contribute to the high mortality rate associated with EOC. These include the unavailability of reliable and accurate early detection markers, late diagnosis at an advanced stage of the disease, limited treatment options, drug resistance, and high recurrence rate [7]. Given the complexities linked to EOC, it is important to explore different treatment options and develop new and effective strategies to tackle the disease. This particular subtype of ovarian cancer heavily relies on elevated glucose metabolism and lactate transport, which hold the potential to serve as the key therapeutic targets [8]. The potential role of MCTs in ovarian cancer pathogenesis is still not well characterized; hence, we aim at exploring the connection between MCTs and different features of cancer progression.

**Figure 1.**
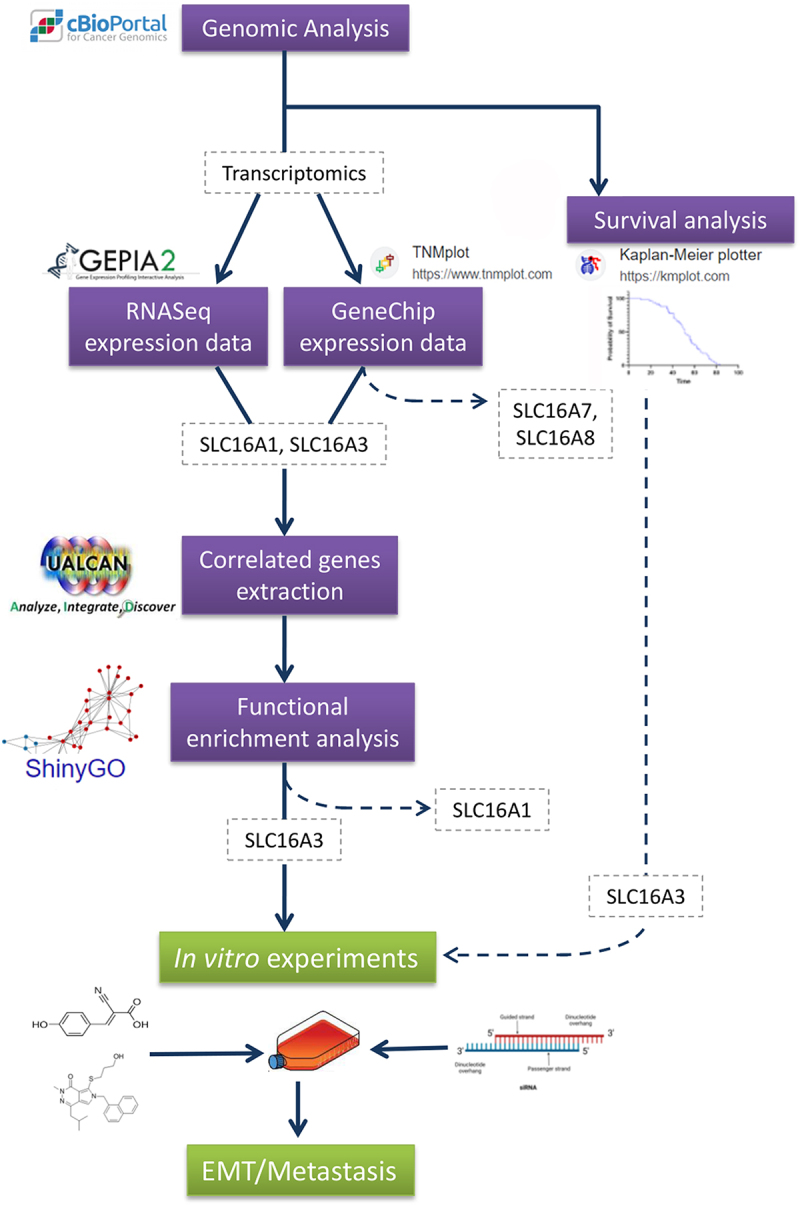
Workflow depicting the experimental design.

With the advent and technological advancements in the field of bioinformatics, it has become extremely easy to access cancer data repositories and perform sophisticated analyses effortlessly. We leveraged quite a few bioinformatics tools to build our hypothesis, before conducting *in vitro* experiments. In this study, we endeavored to associate the expression of the four SLC16A genes with different aspects of ovarian cancer progression. We investigate genomic-level alterations, gene expression, survival status, and metastatic potential associated with functional MCTs in ovarian cancer across different publicly available databases and web tools, to reveal potential candidates among them having the most significant role in terms of cancer progression. Initially, all four genes were considered for the *in silico* analysis, and through a process of elimination only SLC16A1 and SLC16A3 were selected for the gene enrichment analysis. SLC16A3 demonstrated a greater association with oncogenic properties and was validated in ovarian cancer cell lines. Our entire workflow is depicted in Figure 1. SLC16A3 has been shown to have a greater effect on migration and invasion, and thus its gene product MCT4 could be a potential target for cancer therapeutics.

## Materials and methods

### Genomic level alteration using cBioportal

The cBioPortal (http://www.cbioportal.org/) is an online tool used for the analysis of large-scale cancer genomic datasets, and it was used to analyze the DNA-level alteration frequencies of the genes – SLC16A1, SLC16A3, SLC16A7, and SLC16A8 [9,10]. The analysis was performed spanning through all of the TCGA (The Cancer Genome Atlas) tumor data as well as only the Ovarian Serous Cystadenocarcinoma (OSC) data (TCGA, PanCancer Atlas) which had copy number alterations and mutations. Ovarian Serous Cystadenocarcinoma (OSC) is a type of EOC that accounts for the majority of Ovarian cancers. In order to investigate the co-expression of the transporters with respect to EMT (Epithelial–mesenchymal transition) markers, the CPTAC (Clinical Proteomic Tumor Analysis Consortium) dataset for OSC was used in the cBioPortal.

### Transcript level expression data collection from GEPIA2

GEPIA2 (Gene Expression Profiling Interactive Analysis) (http://gepia2.cancer-pku.cn/#index) is a web application exclusively used for genes expression analysis using the huge collection of tumor and normal samples data available in TCGA and the GTEx (Genotype-Tissue Expression) repositories [11]. This web application was used to extract the mRNA expression data of the four SLC16A genes concerned, in the Ovarian cancer samples in TCGA dataset (TCGA_OV) with respect to TCGA normal and GTEx tissue samples marked as control. The expression of all four genes, both in Transcript Per Million (TPM) and log-transformed (log_2_(TPM +1)), was checked between tumor and normal samples, where statistical significance was calculated using ANOVA. q-value and p-value <0.05 were considered significant, for TPM and log-transformed expression data, respectively.

### Differential gene expression analysis for tumor, normal, and metastatic tissues using TNMplot

Another web-based tool for gene expression analysis is the TNMplot (https://tnmplot.com/analysis/), that not only uses tumor and normal tissue data but metastatic tissue data as well; and analyzes both RNA-Seq and GeneChip data [12]. For the comparison of differential gene expression between normal and ovarian cancer tissues, the RNA-Seq module was used while for comparing metastatic tissue with that of the two, the GeneChip module was used. To calculate the statistical significance of RNA-Seq data, Mann–Whitney test while for GeneChip data Kruskal–Wallis test was performed. Additionally, for pair-wise significance testing among tumor, normal, and metastatic data Dunn’s test was implemented by the tool (Supplementary Table S1).

### In silico survival analysis using the Kaplan-Meier plotter

The Kaplan–Meier curve is well known for the estimation of survival functions and gives a probability of survival for a particular individual. Using this idea, the Kaplan-Meier Plotter (https://kmplot.com/analysis/) predicts the correlation between the expression of genes and survival, utilizing the huge amount of data available from TCGA, GEO (Gene Expression Omnibus), and EGA (European Genome-Phenome Archive) databases [13]. This webtool was used to assess the prognostic values of the four SLC16A genes for Ovarian cancer patients using the Pan-cancer RNA-Seq module. The trends for both, the overall survival and the recurrence-free survival, for each of the four genes were checked.

### Acquiring positively correlated genes with that of SLC16A genes in ovarian cancer dataset

The UALCAN (http://ualcan.path.uab.edu) is an interactive web resource for cancer OMICS data analysis and from which all the genes that were positively correlated and statistically significant with SLC16A1 and SLC16A3, were extracted from the TCGA_OV dataset [14]. A Pearson Correlation Coefficient (PCC) ≥ 0.3 was maintained in the obtained set of positively correlated genes. Only two out of the initial four SLC16A genes were selected for this analysis as they showed a higher association with Ovarian cancer in all the previously mentioned analyses. The entire list of the correlated genes has been provided in additional file 1.

### Downstream functional enrichment analysis

The positively correlated gene sets along with the two respective SLC16A genes (as mentioned previously) were then used for functional gene enrichment analysis using ShinyGO (version 0.77) (http://bioinformatics.sdstate.edu/go/) [15], which is a web application that not only represents functional analysis outcomes in different forms and also has access to different pathway databases including Gene ontology GO [16,17] and KEGG (Kyoto Encyclopedia of Genes and Genomes) [18–20]. For this analysis in ShinyGO v0.77, an FDR cutoff of 0.05, pathways size range of 2 to 2000 was used. Also, the top 30 significant pathways, sorted by fold enrichment, from all the mentioned databases were selected for representation, while the complete sets of enriched pathways have been provided in additional file 2.

### CellMiner cross database mining

CellMiner Cross Database (https://discover.nci.nih.gov/rsconnect/cellminercdb), which is a database that helps to study molecular and pharmacological data specifically for cancer cell lines, was used to check the correlation of transcript levels of four SLC16A genes to different lactate levels [21]. Univariate analysis was performed where the X-axis contained the metabolite lactate and the Y-axis showed mRNA levels of different SLC16A genes. The Broad Institute Epithelial ovarian cancer (EOC) cell lines were selected from the tissues module. Lactate (mtb) from the metabolite module and SLC16A genes (exp) from the mRNA expression module of CellMiner CBD (Cell line set- CCLE-Broad-MIT) were used for this analysis. The two cell lines used in the in-vitro studies were highlighted in red.

### Cell culture

SKOV-3 (ATCC, USA) and OAW-42 (Sigma-Aldrich, USA), two human epithelial ovarian cancer (EOC) cell lines were used for the *in vitro* experiments. These cell lines were cultured in RPMI (Gibco) and Dulbecco’s modified Eagle’s medium high glucose (Gibco), respectively; both were supplemented with 10% FBS, 100 µg/mL streptomycin, and 100 U per mL penicillin (Gibco). The cell lines were maintained in a humidified incubator at 37°C with 5% CO_2_. Both cell lines are STR (Short Tandem Repeat) profiled and authentic. MCT1 Inhibitor, SR13800 (Calbiochem, Cat# 509663, Merck Millipore), and α Cyano Hydroxy Cinnamic Acid (α-CHCA) (Sigma, Cat#C2020) were used at a final concentration of 5 nM and 1 mM. For vehicle control, we used DMSO (Sigma).

### Transient transfection

The cells were transfected with pooled siRNA against MCT4 (Santa Cruz MCT4 siRNA (h2): Cat#sc -45,892) at a final concentration of 20 nM using lipofectamine RNAimax (ThermoFisher Scientific, Cat#13778150). The transfection medium Optimem was replaced with the complete medium after 5 h of transfection. The cells were harvested after 72 h of transfection. For control, we have used SiRNA control (Santa Cruz: Cat#sc -37,007).

### Wound-healing assay

Changes in cell migration *in vitro* as an effect of different inhibitors or silencing a gene were studied by wound healing assay. After growing the cells up to the desired confluency, a scratch was made with a 10 µL microtip and at different time points images of the wound were taken with an inverted microscope (EVOS, ThermoFischer Scientific). The images at different time-points were analyzed using ImageJ software. The wound area was represented as a fold of the initial wound area, and the wound closure rate was measured after 24 h and was represented as bar charts. Three independent experiments were conducted for statistical significance.

### L-lactate assay

L-lactate from the conditioned media (CM) of the cells was measured using the Cayman L-Lactate assay kit using the manufacturer’s protocol (Cayman Cat #700510). The protein estimation was done to normalize the lactate concentration. The experiment was done in triplicate.

### Western-blot

Cells were lysed in RIPA lysis buffer, the whole protein was isolated using the standard protocol, and the proteins were then estimated by the Lowry method and resolved in 10% SDS-PAGE. 80 μg protein samples were prepared and were subjected to immunoblotting with antibodies specific for target proteins. For blocking, we used 3% BSA (SRL, Cat#83803) in the TBST buffer. Protein bands were visualized by reacting horseradish peroxidase-labeled secondary Antibodies (ABclonal; 1:5000) with the ECL substrate (Bio-Rad, Cat# 170–5061) by chemiluminescence. Antibodies were used against MCT4 (Santa Cruz, Cat#SC-376140) at 1:500 dilution, MCT1 (Santa Cruz, Cat#SC-365501) at 1:500 dilution, Vimentin (Cell Signaling Technology, Cat#5741) at 1:4000 dilution and E-Cadherin (Santa Cruz, Cat# SC-7870) at 1:2000 dilution. For Secondary HRP-tagged antibodies, we have used HRP Goat anti-mouse (ABclonal, Cat#AS003) and HRP Goat anti-rabbit (ABclonal Cat #AS014). The band intensity was determined using ImageJ software and was normalized with the intensity of α-Tubulin (Cell Signaling Technology, Cat#2144). Relative fold change was plotted from three replicates.

### Invasion assay

24-well inserts with Matrigel-coated Transwell membranes (Corning, Cat#354480) were used to check *in vitro* invasion of the cells. In short, nearly 2.5 × 10^5^ cells were seeded in serum-free media in the upper chamber and were allowed to invade for 22 h to the serum containing lower chamber. Invaded cells in the serum-facing part of the membrane were fixed in ice-cold methanol and subsequently stained in 0.1% crystal violet and counted under the microscope. Cells per field were calculated, and relative invasiveness (fold change of cells invaded) was calculated with respect to control and plotted in bar charts.

### MTT assay for cell viability

Nearly 3000 cells were seeded per well of a 96-well plate and subjected to treatments at different concentrations. After 24 h of the treatment, the medium was discarded and replenished with 10 μl of 5 mg/ml MTT (Invitrogen, Cat#M6494) mixed with 90 μl of serum-free medium per well. The plate was kept in the dark at 37^ο^C. The reaction was terminated after 4 h with DMSO, and the absorbance was measured at 570 nm. The cell viability percentage was calculated as (Treated/Control) *100.

### Actin stress fiber formation analysis by microscopy

Cells were fixed with 4% paraformaldehyde and stained with Phalloidin Rhodamine conjugate (Invitrogen, Cat# R415) after respective treatments and were visualized under the confocal microscope from Leica (TCS SP8, USA) after counterstaining with DAPI (0.33 μg/ml, SRL, Cat#18668). The images were processed using LasX software. For F-actin quantitation, the line intensity profile was drawn by creating a line across the cells using ImageJ software. The number of stress fibers was calculated by counting the number of peaks, and high-intensity stress fibers were considered to have a Fluorescence Intensity of ≥ 20 [22,23]. The nuclear actin stress fibers were calculated by counting the number of overlapping stress fibers within the DAPI channel. The number of high-intensity stress fibers per cell was plotted for representation. The percentage of nuclear actin stress fiber was calculated using the formula (nuclear actin stress fibers/Total number of stress fibers in the cells) *100 and represented as bar charts. The experiments were repeated thrice.

#### Real-time PCR

Real-time PCR was performed for checking mRNA-level expression of MCT4 and MCT1 upon MCT4 knockdown. In brief, the total RNA pool from the cells was extracted using RNA-isoplus (TakaRa Bio, Cat #9109) and subsequent phase separation with Chloroform and precipitation with isopropanol. iScript (BioRad, Cat #1708891) cDNA synthesis kit was utilized to prepare cDNA from total RNA. Real-time PCR was performed in Applied Biosystems, 7500 Real-Time PCR machine, using SYBR green supermix iTaq (Bio-Rad, Cat #1725120). Gene expression was calculated using the ^ΔΔ^CT method using 18S as endogenous control. The primer sequences are provided in Supplementary Table S2.

### Statistical analysis

Apart from the statistical methods used for the *in silico* aspect of the study, GraphPad Prism software (version 8) was used to perform the rest of the statistical significance testing. A Paired t-test (two-tailed) was performed for comparing two groups while for comparing more than two groups, ANOVA was used with Bonferroni’s post hoc test. A p-value <0.05 was considered significant throughout the study unless otherwise stated.

## Results

### Genomic-level alterations of the four functional SLC16A genes

**Figure 2.**
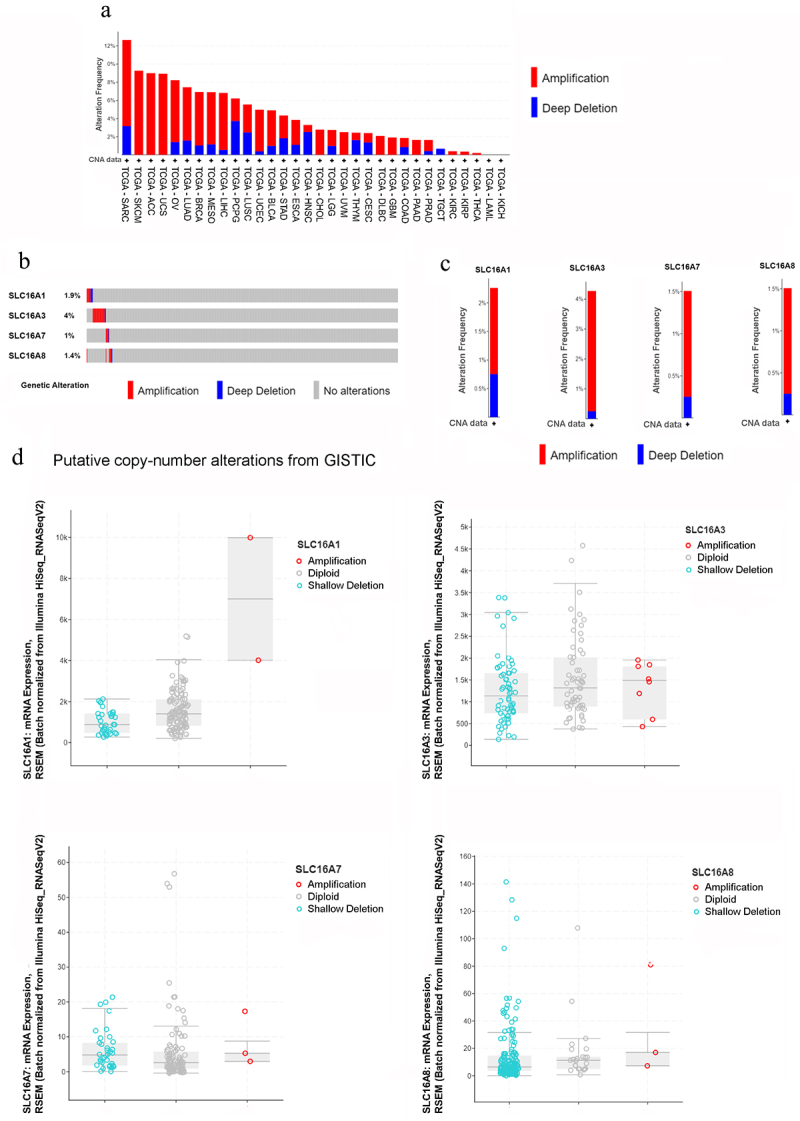
Genomic level alterations of MCTs (SLC16A1/3/7/8) as observed from cBioportal.

Genomic-level alterations often play an important role in regulating protein expression and function. Therefore, we first resorted to find any changes at the DNA level for the four functional MCT genes. When checked at a genomic level for only copy number alterations, the four genes, namely, SLC16A1, SLC16A3, SLC16A7, and SLC16A8, cumulatively show alterations in all the cancers present in the TCGA dataset (TCGA, Pan Cancer Atlas) and Ovarian cancer ranks 5^th^ in that list, as shown in Figure 2a. These four genes are modified at the genomic level in 8.22% of the 572 cases in total that contained copy number alterations, in all the OSC samples (Figure 2b). Among the 398 samples which had mutation along with copy number alterations, in ovarian cancer, SLC16A3 has the highest percentage of alteration (4%) in the patient population and is followed by SLC16A1 comprising 2.5%; whereas SLC16A7 and SLC16A8 are the least altered genes at DNA level among all four functional members of the SLC16A family (Figure 2c). Copy number alteration frequencies of these four genes reveal that SLC16A1 has the highest percentage of deep deletion (0.75%), while SLC16A3 has the highest percentage of amplification (4.02%). Although genomic-level alterations were found in the SLC16A genes in Ovarian cancer samples from TCGA dataset, no significant correlation was observed between copy number amplification and transcript levels in all four genes (Figure 2d).

a. Pan-cancer DNA copy number alteration graph for the SLC16A1/3/7/8 genes where red represents amplification and blue stands for deep deletion.

b. Oncoprint of genomic amplification and deep deletion of SLC16A1/3/7/8 genes in ovarian cancer (TCGA-OV) containing all 572 Ovarian cancer samples with only copy number alterations

c. A visual summary of DNA copy number alteration of the individual SLC16A1/3/7/8 genes in ovarian cancer (TCGA-OV) dataset of 398 samples having both mutation and copy number alterations

d. Boxplot representation of mRNA expression of the SLC16A1/3/7/8 genes vs putative copy number alteration of the same in TCGA-OV dataset of 398 samples containing both mutation and copy number alterations

### Transcript level alterations of the functional SLC16A genes in ovarian cancer patients

**Figure 3.**
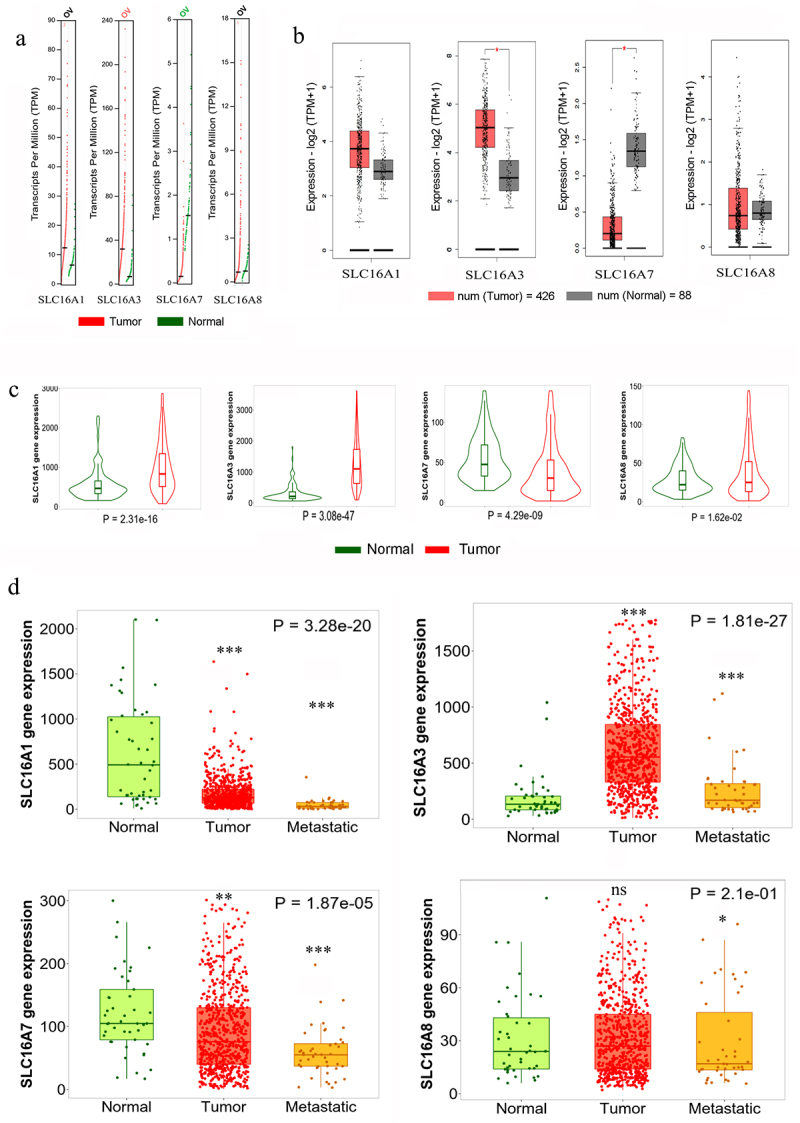
Transcript level alterations of SLC16A1, SLC16A3, SLC16A7, SLC16A8 in ovarian cancer tissue.

DNA-level copy number gain or loss did not necessarily influence the transcript levels of the genes; thus, GEPIA2 was used to assess the transcript-level data of the four SLC16A genes in Ovarian cancer, which uses the RNA-Seq data from TCGA-OV cohort as well as the mRNA-level data of normal ovarian tissue from both TCGA-OV cohort and GTEx datasets. As depicted in Figure 3(a,b), SLC16A1 mRNA was non-significantly increased in Ovarian cancer tissues, while SLC16A3 showed a significant increase for the same. A significant decrease in the transcriptional level was observed for SLC16A7 in the cancer tissues, while a non-significant decrease persisted in the case of SLC16A8. Using the RNA-Seq data available in the TNMplot webtool, similar trends in the expression profiles for these genes were also observed (Figure 3c). Here, not only SLC16A3 but also SLC16A1 showed a statistically significant elevation of its mRNA level in the OC tissues. When the GeneChip dataset in TNMplot was compared, SLC16A3 was found to increase significantly in the tumor and metastatic tissues in Ovarian cancer samples as compared to normal tissues, while other members showed a significant decrease in metastatic tissues (Figure 3d). SLC16A3 levels in tumor vs. metastatic tissues showed non-significant changes (Supplementary table S1).

a. Expression profile of SLC16A1/3/7/8 mRNA (represented in Transcripts Per Million or TPM) in normal (GTEx+ TCGA) depicted in green vs Ovarian Cancer tissue (TCGA-OV) shown in red from GEPIA2.

b. Boxplot representation of SLC16A1/3/7/8 mRNA expression profile (in log TPM) for normal (GTEx + TCGA) vs Ovarian Cancer tissue (TCGA-OV) from GEPIA2. *p < 0.05

c. Violin plot for gene expression (RNA-Seq) profile for SLC16A1/3/7/8 genes from TNMplot comparing normal vs ovarian cancer tissues.

d. Boxplot representation for gene expression data (GeneChip) for SLC16A1/3/7/8 from TNMplot comparing normal, ovarian malignant tumor, and metastasis tissues. The statistical significance between normal and tumor/metastatic samples was calculated using Dunn’s test. *p < 0.05, **p < 0.01, ***p < 0.001, ns = non-significant

### Prognostic values of mRNA level of the four SLC16A genes in ovarian cancer patients

**Figure 4.**
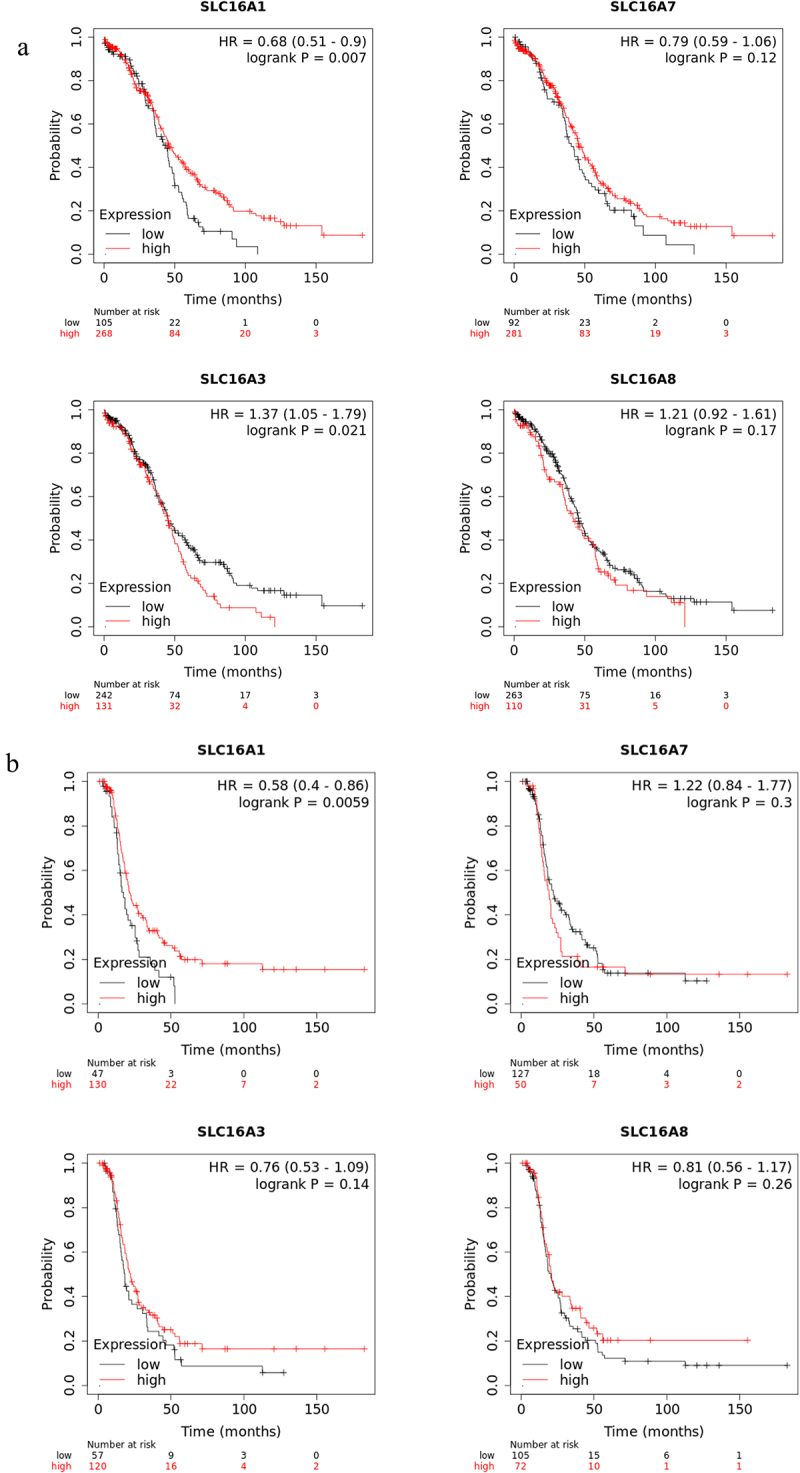
Prognostic value of the SLC16A1/3/7/8 genes in ovarian cancer from Kaplan-Meier plotter.

Kaplan–Meier plotter was used to analyze the prognostic values of the SLC16A genes in Ovarian cancer patients. Higher expression of SLC16A3 or MCT4 (HR or Hazard Ratio 1.37) was found to be significantly (*p* = 0.021) associated with poor Overall Survival (OS) in Ovarian cancer patients (Figure 4a), although no significant contribution was observed for Recurrence-Free Survival (RFS) (Figure 4b). For RFS as well as OS, higher expression of SLC16A1 was associated with better survival in ovarian cancer patients (HR 0.68, *p* = 0.007 for OS, HR 0.058, *p* = 0.0059 for RFS), while SLC16A7 and SLC16A8 did not show any significant relation with either OS or RFS in ovarian cancer patients. Overall, this suggests the potential role of increased SLC16A3 expression levels in reducing the life expectancy of Ovarian cancer patients. Opposed to that, increased SLC16A1 does not act as a poor prognostic marker for Ovarian cancer.

a. The Overall Survival of Ovarian cancer patients associated with mRNA levels of the SLCA16 genes.

b. Recurrence-Free Survival of Ovarian cancer patients associated with mRNA levels of the SLCA16 genes.

### Gene-association study for SLC16A1 and SLC16A3

**Figure 5.**
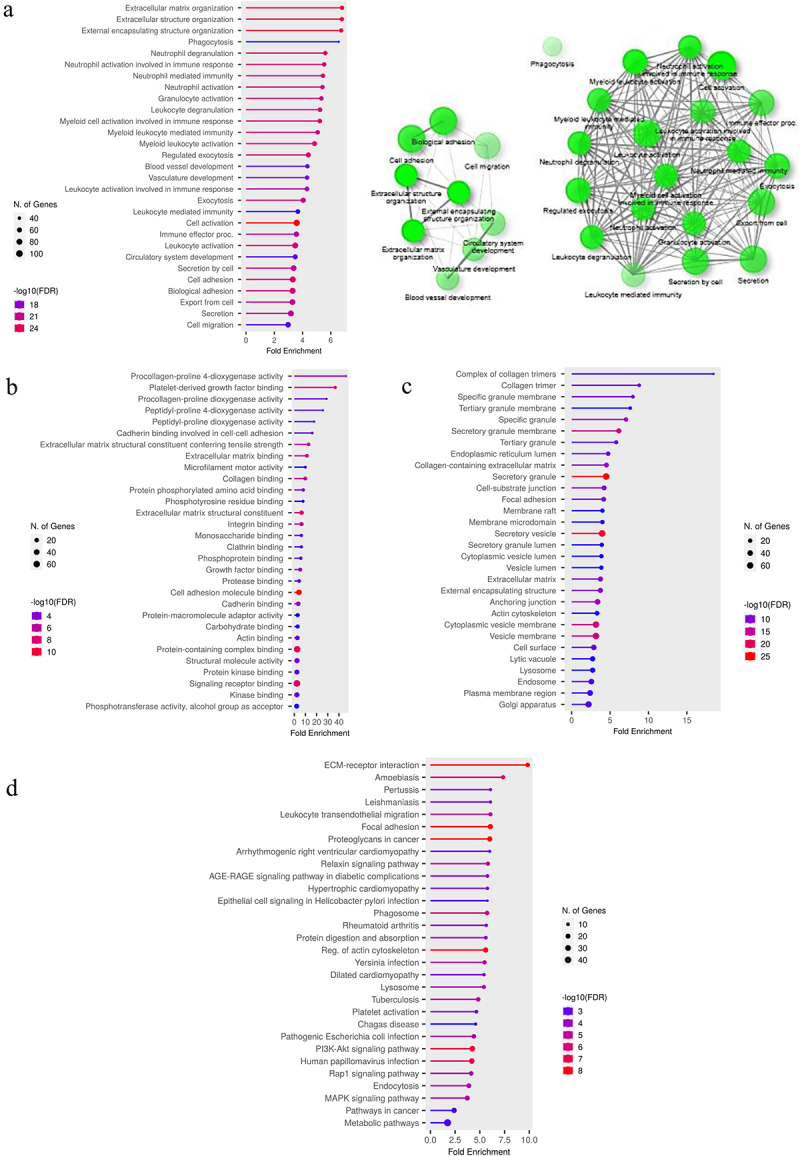
Pathway enrichment analysis for the SLC16A3 gene in TCGA-OV.

Although SLC16A3 was found to be associated with poor patient outcomes in ovarian cancer, several studies have indicated the same trend for both SLC16A1 (MCT1) and SLC16A3 (MCT4) in different cancers [4,24,25]. In this study also increased expression of these two genes was observed, from RNA-Seq analysis in both GEPIA2 and TNMplot. Even though the GeneChip data from TNMplot demonstrated a reverse trend with that of RNA-Seq results for SLC16A1, the RNA-Seq analysis generally has been found to be more comprehensive, sensitive, and reliable [26,27], and thus we inclined toward RNA-Seq outcomes. Consequently, the gene families and the molecular functions associated with these two genes in the ovarian cancer tissues were examined. To evaluate the functional enrichment of SLC16A1 and SLC16A3 associated genes in the Ovarian cancer dataset, the gene sets acquired from UALCAN were used for enrichment analysis in ShinyGO (v0.77), using both GO and KEGG pathways. Gene set correlated with SLC16A3 showed association with different biological processes like extracellular matrix organization, extracellular structure organization, neutrophil degranulation, extracellular matrix binding, and so on in the GO biological processes (Figure 5a), molecular function (Figure 5b) and cellular component (Figure 5c) databases. KEGG enrichment analysis for SLC16A3 (Figure 5d) showed an association with leukocyte trans-endothelial migration, regulation of actin cytoskeleton and ECM receptor interaction, indicating its potential role in tissue invasion and metastasis [28,29]. Apart from this, the geneset positively correlated with SLC16A1 was also found to be associated with different biological functions. Findings are included in Supplementary Figure 1. There were several pathways that were shown to be associated with the given genes, but we only have focused on the ones that have known association with cancer progression (Supplementary tables 3–6). Thus, SLC16A3 was selected for all downstream *in vitro* experiments, to demonstrate the validity of a few of the aforementioned biological associations.

a. The top 30 significant pathways of Gene Ontology (Biological pathway), associated with SLC16A3 gene and sorted by fold change, are represented along with the network of closely related pathways.

b. Gene Ontology (Molecular function) and c. Gene Ontology (Cellular component) enrichment analysis showing the top 30 pathways, sorted by fold change, that are associated with SLC16A3 gene.

d. KEGG pathway enrichment analysis for genes correlated to SLC16A3 gene and representation of top 30 pathways.

### Effect of MCT4 blocking in vitro on the metastatic potential of the ovarian cancer cells

**Figure 6.**
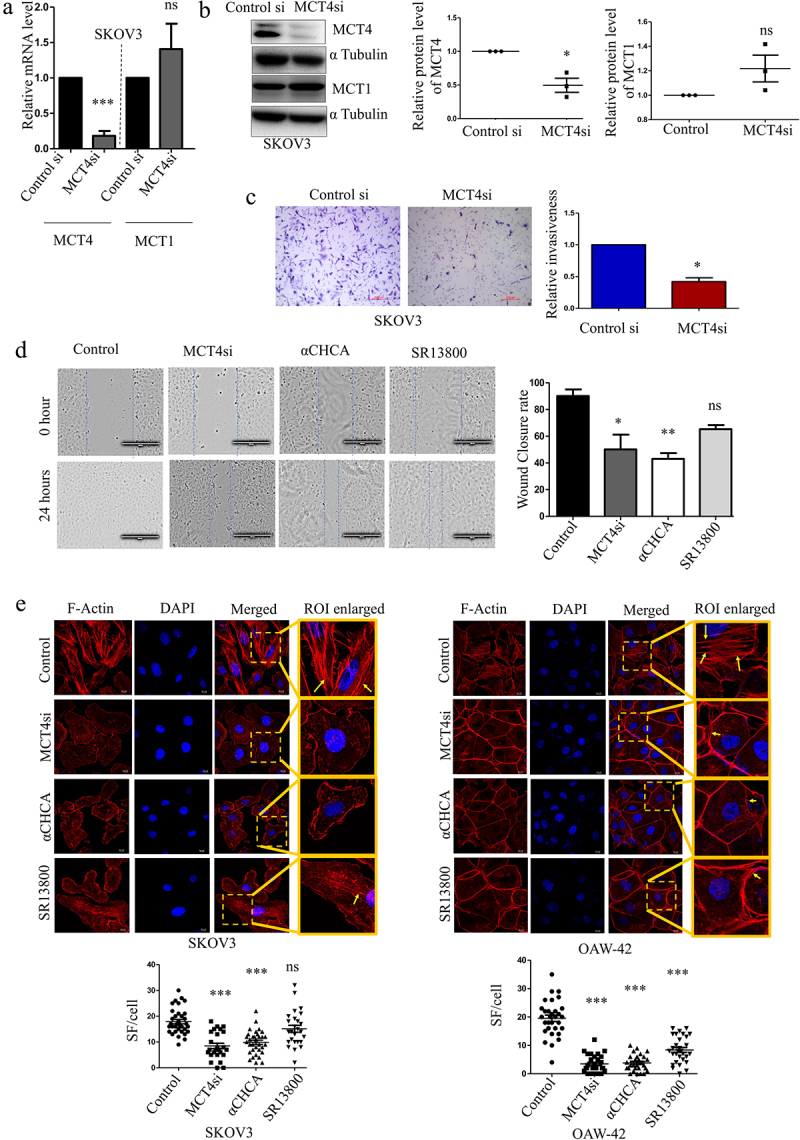
Effects of MCT4 blocking on metastatic features of the ovarian cancer cells.

Since, we found the association of MCT4 (SLC16A3) with different functions related to metastasis as well as its effect on poor prognosis in the patients, we tried to find if blocking MCT4 can be helpful in mitigating such phenotypes. To validate our *in silico* findings, we have used cell lines which represent the similar type of Ovarian carcinoma as TCGA-Ovarian Cancer dataset and since MCT4-specific blocker is not available yet, we have tested the effects using a siRNA mediated knockdown approach. Realtime PCR was performed to check if the MCT4 knockdown could reduce MCT4 mRNA levels without altering the MCT1 levels. In SKOV3 cells, MCT4-mediated transient knockdown did not reduce the MCT1 levels, but rather increased the transcript level of MCT1 in a non-significant manner (Figure 6a). Transient MCT4 silencing reduced MCT4 protein levels significantly in SKOV3 and OAW-42 cells (Figure 6b, Supplementary Figure S2A) which represent two Ovarian Adenocarcinoma cell types [30]. MCT4 knockdown by siRNA mediated transfection decreased the invasion of the SKOV3 cells through Matrigel invasion chambers as observed from the Matrigel invasion assay (Figure 6c). By comparing the effects of MCT1/2 blocker SR13800 [31,32] and a generalized MCT blocker α-Cyano Hydroxy Cinnamic Acid (α-CHCA) [4,33] to MCT4 silencing in SKOV3 cell line, decreased migration rate was observed in all three compared to DMSO-treated control cells in wound healing assay (Figure 6d). The cytotoxicity associated with these two drugs was also assessed through the MTT assay (Supplementary Figure S2B, C). Regulation of the actin cytoskeleton emerged as one of the enriched terms associated with MCT4 in the KEGG pathway, as depicted in Figure 5d. Stress fiber formation, a particular type of actin cytoskeletal modifications required in the invasive cells, was decreased significantly after transient MCT4 silencing in both SKOV3 and OAW-42 cells (Figure 6e) as determined through phalloidin staining of F-actin and visualized by confocal microscopy [34]. Treatment with 1 mM α-CHCA also was found to drastically reduce the number of high-intensity stress fibers per cell in both cell lines (Figure 6e). MCT1/2 blocker SR13800 treatment reduced F-actin formation significantly in the OAW-42 cell line, while non-significant changes were observed in SKOV3 (Figure 6e). The nuclear F-Actin percentage did not show any significant variations across different treatment groups (Supplementary Figure S2D) though the total number of stress fibers was reduced. These experiments clearly demonstrate the impact of using MCT blockers in regulating different metastatic features of the EOC cells.

a. Validation of MCT4 siRNA in SKOV3 cells by real-time PCR. The relative RNA levels of MCT4 and MCT1 upon MCT4 knockdown (72 h) normalized to 18S.

b. Western blot and histogram showing the relative level of MCT4 and MCT1 proteins upon MCT4 siRNA mediated knockdown in SKOV3. Observed Molecular weight of MCT4, MCT1, and αTubulin are 44KDa, 40KDa and 55KDa, respectively.

c. *In vitro* study of SKOV3 cells in MCT4 knockdown condition showing reduced invasiveness due to Matrigel invasion assay.

d. Reduced migration rate in MCT4 knockdown conditions and in presence of MCT blockers α-Cyano Hydroxy Cinnamic Acid (α-CHCA) and SR13800 compared to control SKOV3 cells (Adjusted *P* Value for Control vs. MCT4si is 0.0119, Control vs. α-CHCA is 0.0046 and Control vs. SR13800 is 0.1107).

e. Comparison of High-intensity Stress-Fiber (SF) upon MCT blocking (adjusted *P* value for Control vs. MCT4si is <0.0001, Control vs. α-CHCA is <0.0001, and Control vs. SR13800 is 0.2784 for SKOV3). The adjusted *P* values for OAW-42 for the same are <0.0001 for all. Arrows indicate the stress fibers. *p < 0.05, **p < 0.01, ***p < 0.001, ns = non-significant.

### MCT4 blocking reverses the epithelial to mesenchymal transition of the ovarian cancer cells

**Figure 7.**
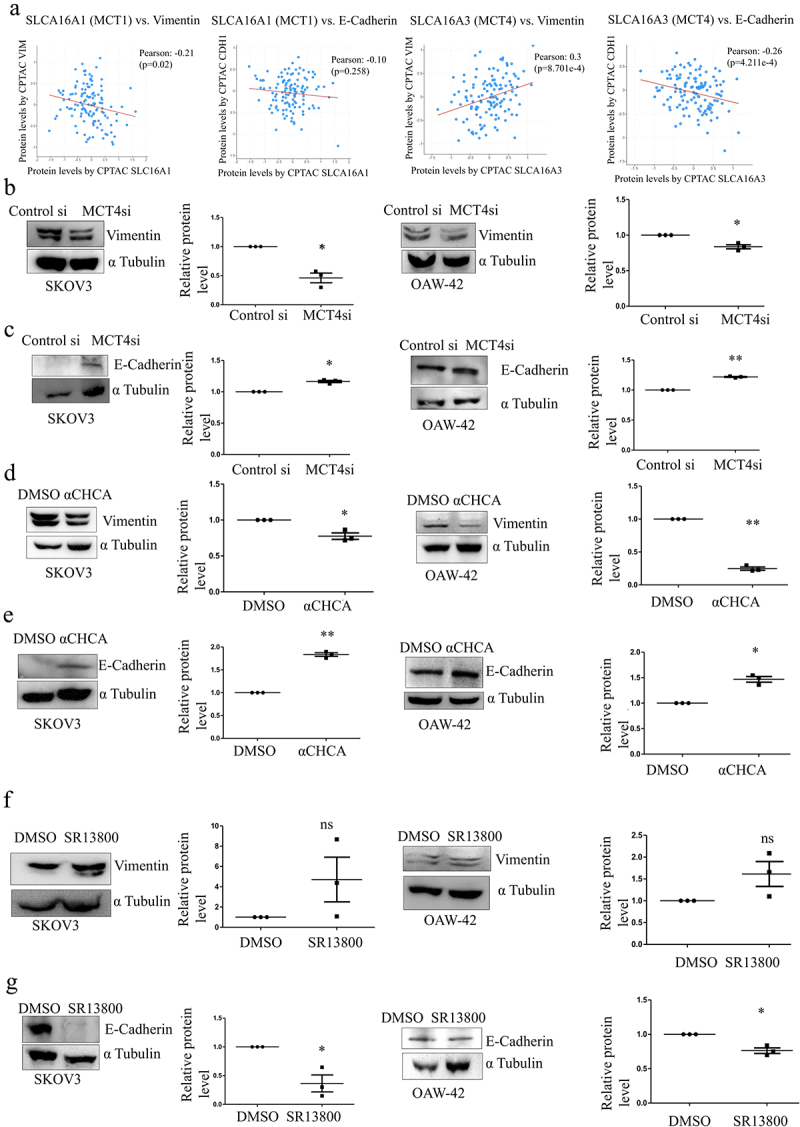
Expression of EMT markers on MCT blocking in ovarian cancer cells.

Metastasis requires mesenchymal changes in the epithelial cancer cells, which are characterized by higher expression of mesenchymal markers like vimentin (VIM) and a decrease in the epithelial markers like E-cadherin (CDH1). From the ovarian cancer dataset of CPTAC, which concerns the protein-level data in the C-BioPortal, a positive correlation was found between MCT4 and Vimentin (PCC 0.3 and p-value 8.70e^−4^), while a negative correlation existed between MCT4 and E-Cadherin (PCC −0.26 and p-value 4.211e^−3^) as represented in Figure 7a. Similarly, MCT4 silencing resulted in reduced Vimentin in both SKOV3 and OAW-42 cells (Figure 7b), while E-Cadherin was increased (Figure 7c). 1 mM α-CHCA treatment had similar consequences as MCT4 knockdown (Figure 7d−e). MCT1/2 blocker SR13800 treatment failed to reverse the EMT phenotype of the cells (Figure 7f−g) proving the immense importance MCT4 holds in regulating the EMT phenotype and subsequent invasive behavior of the cells.

a. Correlation of Vimentin and E-Cadherin to MCT1 and MCT4 from CPTAC database of Ovarian cancer.

b. Western Blot analysis showing reduced expression of mesenchymal marker Vimentin (57KDa) upon MCT4 silencing.

c. Elevated protein level of Epithelial marker E-Cadherin (97KDa) upon MCT4 knockdown.

d. Relative expression of Vimentin upon α-CHCA treatment on Ovarian cancer cells.

e. Western blot analysis showing changes in the expression of E-Cadherin upon α-CHCA treatment.

f. Vimentin protein levels upon MCT1/2 blocker SR13800 treatment in EOC cell lines.

g. Decreased E-Cadherin levels upon administration of MCT1/2 blocker SR13800. *p < 0.05, **p < 0.01, ***p < 0.001, ns = non-significant

### Impact of MCT targeting on lactate levels

**Figure 8.**
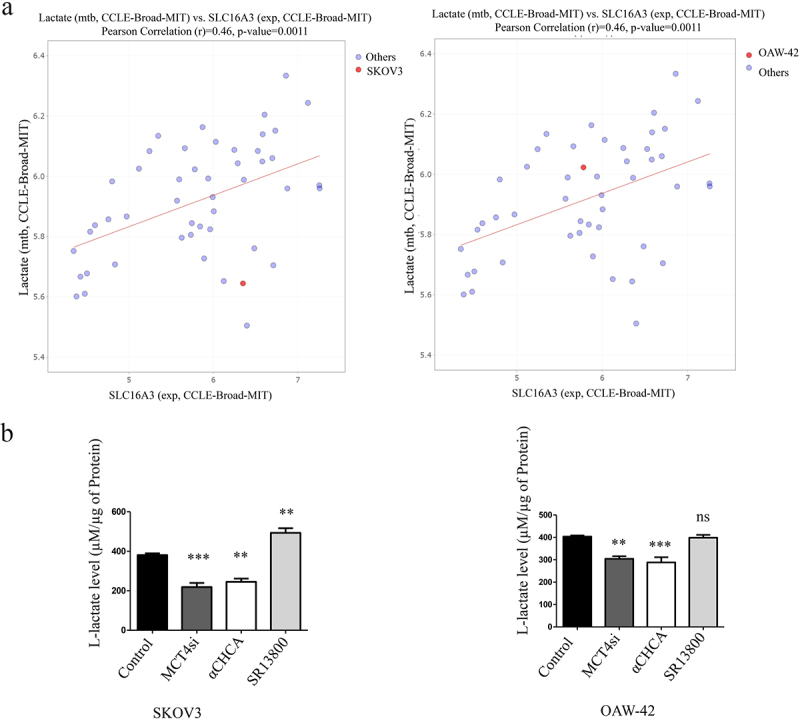
Fate of extracellular lactate concentration upon MCT blocking.

There is a high lactate accumulation in the Tumor Micro-environment or TME, which mediates cancer cell invasion and metastasis, so, we checked if nonspecific MCT blocking by α-CHCA can reduce the lactate secretion from the glycolytic cancer cells. We examined the correlation between lactate and expression of MCTs in different OC cell lines (Broad-MIT CCLE) including SKOV3 and OAW-42 (Figure 8a), Supplementary Figure S3). A statistically significant positive correlation was observed for MCT4 (PCC 0.46, *p* = 0.0011) and MCT3 (PCC 0.42, *p* = 0.0033). A similar reduction in extracellular L-lactate was found between α-CHCA treated group alongside MCT4 silencing, while MCT1/2 blocking could not effectively reduce the extracellular lactate levels (Figure 8b). While SR13800 treatment significantly increases the extracellular lactate levels in the SKOV3 cells, it does not significantly alter the extra-cellular lactate levels in OAW-42 cells. This indicates that lactate export was perturbed when MCT4 was targeted either by pharmacological blocking using α-CHCA or through siRNA mediated knockdown.

a. Correlation of lactate levels to MCT4 (SLC16A3) mRNA in Epithelial Ovarian cancer cell lines of CCLE (Broad Institute).

b. Relative lactate levels upon different MCT blocking *in vitro.*

(Adjusted *P* Value for Control vs. MCT4si is 0.0003, Control vs. α-CHCA is 0.0014 and Control vs. SR13800 is 0.006 for SKOV3 cells; The same for OAW-42 is 0.0019,0.0004 and >0.9999 for Control vs. all the groups, respectively)

*p < 0.05, **p < 0.01, ***p < 0.001, ns = non-significant

## Discussion

The morbidity of ovarian cancer remains high till date due to its complex molecular signatures, lack of specific biomarkers, high metastatic rate, and also less treatment options. Due to these complexities, it is important to look into alternative therapeutic aspects associated with the disease [6]. The highly glycolytic nature of this cancer can put forth a potential therapeutic feature where monocarboxylate transporters may serve as ideal targets as well as can pose as important prognostic factors in the case of ovarian cancer. Our study evaluates the importance of these functional monocarboxylate transporters MCT1, MCT2, MCT3, and MCT4 in Ovarian cancer pathophysiology and their relevance as therapeutic targets.

Different studies of the monocarboxylate transporters reveal their importance in several cancers. MCT1 was found to be a good prognostic marker for endometrial cancer [35] and a subtype of lung cancer [36], while MCT2 was proven to be important as a biomarker in prostate cancer [37]. MCT3 can be explored as a molecular target in a subtype of breast cancers [38] and MCT4 is increased in glycolytic tissues and several cancers including hepatocellular carcinoma [39] and can predict patient outcomes. Different independent studies revealed the pharmacological blocking of different monocarboxylate transporters to be efficient in reducing oncogenic properties like proliferation, migration, invasion, drug resistance, immune cell modulation [40–42]; therefore, it is quite relevant to explore their roles in ovarian cancer progression.

Our study reveals that MCT4 encoded by the SLC16A3 gene shows a high level of genomic alterations in ovarian cancer patients followed by MCT1 encoded by the SLC16A1 gene. But DNA level alterations (amplifications and deletions) do not correlate with mRNA levels. Compared to normal tissues, ovarian cancer tissues showed higher mRNA levels for SLC16A1 and SLC16A3, the two most important lactate transporters. SLC16A3 mRNA was not only increased in metastatic tissues but also was responsible for poor prognosis in the OC patients proving it as one of the major transporters with possible therapeutic intervention. There could be a number of possible ways for poor prognosis in patients. Thus, we tried to look into the genes which are mostly associated with SLC16A3 or MCT4 and their functional implications. We selected the positively correlated genes and subjected them to enrichment analysis to further narrow down the oncogenic properties associated with SLC16A3 in OC. We also did similar gene enrichment studies to find the functions associated with SLC16A1 but most of the enrichment terms were associated with DNA and RNA processing and functions indicating a possibility that the enriched pathways might be present upstream of the gene. It could also mean the association of SLC16A1 with biosynthetic pathways, although no overall oncogenic terms were found except for cell cycle regulation. SLC16A1 was also not found to be associated with poor patient survival. Contrary to that, for SLC16A3, extracellular matrix organization, extracellular structure organization, neutrophil degranulation, extracellular matrix binding, leukocyte trans endothelial migration, regulation of actin cytoskeleton and ECM receptor interaction were some of the functions found to be enriched which in turn implied that the EMT and metastatic functions were associated with MCT4 in OC as well as the immune modifications. These two functions, metastasis and immune invasion are considered two major hallmarks of cancer and play a very important role in cancer progression [43]. But a simple gene association study does not imply that MCT4 is responsible for these functions and can also indicate that these genes might be co-transcribed alongside MCT4. So, in order to validate whether MCT4 is actually responsible for the metastatic phenotype of the cancer cells, we transiently knocked down MCT4 with MCT4 siRNA in ovarian adenocarcinoma cell lines and observed its effects. We indeed found reduced metastatic potential in MCT4 silenced cells. Since MCT1 was found to be non-significantly increased upon MCT4 silencing, the downstream effects observed by the knock-down were probably due to reduced MCT4 levels. EMT or epithelial to mesenchymal transition is associated with metastasis which is characterized by the reduction of Epithelial markers and increased mesenchymal markers alongside increased F-actin polymerization and stress fiber formation. MCT4 reduction also reduced the mesenchymal phenotypes alongside decreasing the high intensity stress fiber formation. Treating the cells with a nonspecific inhibitor for MCTs, α-Cyano Hydroxy Cinnamic Acid or α-CHCA, yielded similar results to MCT4 silencing while using MCT1/2 specific inhibitor SR13800 could only partially reduce certain metastatic phenotypes like migration and F-actin formation but could not reverse the EMT phenotype. This also suggests that the functions of some MCTs could be compensated by other members. Lactate being one of the key metabolites transported out by cancer cells can help in metastasis and we found that SR13800 did not reduce the lactate levels while MCT4 silencing and α-CHCA treatment could reduce extracellular lactate levels showing a possible mechanism behind increased metastasis of the cancer cells associated with MCT4. The slight increase in the extracellular lactate levels upon SR13800 administration might be a result of MCT1/2 specific blockage of lactate import in SKOV3 cells. It was observed that the same treatment failed to effectively reduce the stress fibers in the SKOV3 cells, further confirming the fact that blocking lactate import might not be as efficient as targeting the lactate export. Contrary to some of the findings indicating the potential role of MCT1 in cancer cells, our data demonstrate that in the Ovarian cancer context, blocking MCT1 might not be as effective as MCT4 blocking. However, the present study does not include the immunological functions that might be associated with MCT4 as derived from gene association studies. The tumor microenvironment contains different types of immune cells which play a critical role in cancer progression and in the future, the association between these immune cells with MCT4 expression in cancer tissues could be explored. New therapeutic approaches could be adopted in the future to specifically target MCT4 in Ovarian cancer tissues as a part of different strategies developed for the functional blocking of metabolic transporters. Alongside this, to obtain a deeper understanding of MCT4, the structural and functional aspects of this transporter protein could be studied, via structural analysis and simulations. In addition, focusing on the metabolomics aspect could reveal the changes in the levels of different small molecules and glycolytic intermediates, associated with MCT4 expression in cancer tissues, and not limit our observation to only changes in lactate levels. Furthermore, SLC16A7 was found to exhibit opposing patterns with SLC16A3 in the examined modules. This SLC16A7 gene encodes for MCT2 protein which has the lowest Km (Michaelis constant) or the highest affinity for lactate followed by SLC16A8 (MCT3), SLC16A1 (MCT1) and SLC16A3 (MCT4) [44]. This allows MCT4 to carry out most of the export function, while MCT1 (SLC16A1) can perform both export and import. MCT2 plays a suitable role in regulating only the import of substrates. It has been reported that there is a progressive decrease of MCT2 in hepatocellular carcinoma, while an increase in MCT4 [45]. Since cancer progression heavily relies on increased glycolysis and subsequent lactate export, it has been suggested that the increased expression of MCT4 is in accordance with the metabolic phenotype of the cancer cells. As the cancer cells are adapted more toward glucose utilization instead of glucose synthesis, MCT2 showed decreased expression [45]. Though in this paper, no experiments were performed to prove this reciprocal relationship of MCT2 (SLC16A7) and MCT4 (SLC16A3), this evidence from hepatocellular carcinoma might explain similar observations for Ovarian cancer and could be further validated in future using *in vitro* experiments.

Therefore, our study suggests that MCT4 is associated with a poor prognosis of ovarian cancer and has a strong impact on the metastasis of cancer cells alongside being a key glycolytic transporter.

## Conclusion

The present study reveals the association of functional MCTs with ovarian cancer clinicopathologic features to reveal the potent drug target. MCT4 was found to be associated with ovarian cancer cell metastasis both *in silico* and *in vitro*. Our study includes *in vitro* data derived from cues of the ovarian cancer clinical data to finally reveal the possible therapeutic intervention. Considering the impact of MCT blocking in reversing cancer progression, developing more specific MCT4 blockers in the future might be important in ameliorating ovarian cancer metastasis.

## Supplementary Material

Supplemental Material

## Data Availability

The data presented in this study are included in the article or supplementary information. All the raw files are available on request to the corresponding author. Data extracted from publicly available databases and web tools could be reproduced online.

## Abbreviations

TCGA (The Cancer Genome Atlas), MCT (Monocarboxylate Transporter), OSC (Ovarian Serous Cystadenocarcinoma), OC (Ovarian Cancer), EOC (Epithelial Ovarian Cancer), α-CHCA (α-Cyano Hydroxy Cinnamic Acid), GTEx (Genotype-Tissue Expression), GO (Gene Ontology), KEGG (Kyoto Encyclopedia of Genes and Genomes), EMT (Epithelial to Mesenchymal Transition).
