# Supplementary material for: A systemic analysis of monocarboxylate transporters in ovarian cancer and possible therapeutic interventions
Source: Channels (Austin). 2023 Nov 7;17(1):2273008. doi: 10.1080/19336950.2023.2273008 (PMC10631444; doi:10.1080/19336950.2023.2273008)
Supplement: Supplemental Material [file KCHL_A_2273008_SM4276.zip › Supplementary files/Supplementary Figure caption.docx]

**Supplementary Figure 1**: **Gene association study for** **SLC16A1 in TCGA-OV.**

1. Gene Ontology (Biological pathway) study of SLC16A1 associated genes in Ovarian cancer tissues and representation of the top 30 pathways, sorted by fold change. Closely related pathways are depicted in network.
2. Chart depicting the Gene Ontology (Molecular function) of SLC16A1 associated genes in Ovarian cancer tissues (the top 30 pathways, sorted by fold change)
3. Chart depicting the Gene Ontology (Cellular Component) of SLC16A1 associated genes in Ovarian cancer tissues (the top 30 pathways, sorted by fold change)
4. KEGG pathway enrichment analysis for genes correlated to SLC16A1 in TCGA-OV.

**Supplementary Figure 2: In -vitro assessment of MCT4 knock-down and blocking.**

1. Western blot and quantitation showing effective MCT4 knock-down by siRNA mediated transfection in OAW-42 cells.
2. Bar charts representing viable cell population by MTT assay using α-CHCA at different concentrations.
3. Diagram depicting viable cell population by MTT assay using SR13800 at different concentrations.
4. Nuclear Stress fiber percentage in SKOV3 and OAW-42 cells upon different treatment conditions.

*p<0.05,**p<0.01,***p<0.001, ns=non significant

**Supplementary Figure 3: Correlation of mRNA levels of SLC16A1,7,8 with lactate levels in Epithelial Ovarian cancer cell lines (CCLE- Broad Institute)**
