# Supplementary material for: A systemic analysis of monocarboxylate transporters in ovarian cancer and possible therapeutic interventions
Source: Channels (Austin). 2023 Nov 7;17(1):2273008. doi: 10.1080/19336950.2023.2273008 (PMC10631444; doi:10.1080/19336950.2023.2273008)
Supplement: Supplemental Material [file KCHL_A_2273008_SM4276.zip › Supplementary files/supplementary_tables2_Oct13_2023.docx]

**Supplementary table 1:** Table obtained from TNMplot depicting p-value for pair-wise significance testing using Dunn’s test, among Tumor, Normal and Metastatic samples.

|  | Normal-Tumor | Tumor-Metastatic | Normal-Metastatic |
| --- | --- | --- | --- |
| SLC16A1 | 1.55E-08 | 2.66E-21 | 9.27E-14 |
| SLC16A3 | 2.98E-19 | 1.24E-01 | 4.83E-13 |
| SLC16A7 | 2.36E-03 | 1.70E-06 | 1.95E-04 |
| SLC16A8 | 4.87E-01 | 1.01E-01 | 3.88E-02 |

**Supplementary table 2:** Primer list for Real Time PCR.

| Gene | Forward (5′- 3′) | Reverse (5′- 3′) | Tm |
| --- | --- | --- | --- |
| 18S | GATTCCGTGGGTGGTGGTGC | AAGAAGTTGGGGGACGCCGA | 60 °C |
| MCT4 | GTTGGGTTTGGCACTCAACT | GAAGACAGGGCTACCTGCTG | 60 °C |
| MCT1 | CACTTAAAAATGCCACCAGCA | AGAGAAGCCGATGGAAATGA | 60 °C |

**Supplementary table 3:** Enriched pathways (KEGG) associated with SLC16A3 where cancer associated terms are indicated in **Bold fonts**.

| Enrichment FDR | nGenes | Pathway Genes | Fold Enrichment | Pathway |
| --- | --- | --- | --- | --- |
| 5.22E-09 | 15 | 88 | 9.837169 | **ECM-receptor interaction** |
| 9.06E-07 | 13 | 102 | 7.355374 | Amoebiasis |
| 1.66E-05 | 12 | 114 | 6.074883 | **Leukocyte transendothelial migration** |
| 0.000557539 | 8 | 76 | 6.074883 | Pertussis |
| 0.000557539 | 8 | 76 | 6.074883 | Leishmaniasis |
| 5.22E-09 | 21 | 200 | 6.059696 | **Focal adhesion** |
| 5.22E-09 | 21 | 202 | 5.999699 | **Proteoglycans in cancer** |
| 0.000588381 | 8 | 77 | 5.995989 | Arrhythmogenic right ventricular cardiomyopathy |
| 1.16E-05 | 13 | 129 | 5.815877 | Relaxin signaling pathway |
| 0.0001372 | 10 | 100 | 5.771139 | AGE-RAGE signaling pathway in diabetic complications |
| 0.000345171 | 9 | 90 | 5.771139 | Hypertrophic cardiomyopathy |
| 0.001884011 | 7 | 70 | 5.771139 | Epithelial cell signaling in Helicobacter pylori infection |
| 2.03E-06 | 15 | 151 | 5.73292 | Phagosome |
| 0.00039227 | 9 | 92 | 5.64568 | Rheumatoid arthritis |
| 0.000169179 | 10 | 103 | 5.603048 | Protein digestion and absorption |
| 1.50E-08 | 21 | 217 | 5.584973 | **Regulation of actin cytoskeleton** |
| 1.75E-05 | 13 | 137 | 5.476264 | Yersinia infection |
| 3.96E-05 | 12 | 128 | 5.410443 | Lysosome |
| 0.000525882 | 9 | 96 | 5.410443 | Dilated cardiomyopathy |
| 1.51E-05 | 15 | 179 | 4.83615 | Tuberculosis |
| 0.000620053 | 10 | 124 | 4.654145 | Platelet activation |
| 0.003285794 | 8 | 101 | 4.571199 | Chagas disease |
| 3.62E-05 | 15 | 197 | 4.394268 | Pathogenic Escherichia coli infection |
| 3.80E-08 | 26 | 354 | 4.23869 | **PI3K-Akt signaling pathway** |
| 1.87E-07 | 24 | 331 | 4.184512 | Human papillomavirus infection |
| 6.57E-05 | 15 | 210 | 4.122242 | **Rap1 signaling pathway** |
| 3.76E-05 | 17 | 252 | 3.893229 | Endocytosis |
| 2.10E-05 | 19 | 294 | 3.729648 | **MAPK signaling pathway** |
| 0.001548785 | 22 | 530 | 2.395567 | **Pathways in cancer** |
| 0.001719785 | 46 | 1527 | 1.738523 | **Metabolic pathways** |

**Supplementary table 4 :** Enriched pathways (GO-BP) associated with SLC16A3 where cancer associated terms are indicated in **Bold fonts**.

| Enrichment FDR | nGenes | Pathway Genes | Fold Enrichment | Pathway |
| --- | --- | --- | --- | --- |
| 2.47E-25 | 53 | 450 | 6.79712 | **Extracellular matrix organization** |
| 2.47E-25 | 53 | 451 | 6.782048 | **Extracellular structure organization** |
| 2.57E-25 | 53 | 454 | 6.737233 | External encapsulating structure organization |
| 1.96E-16 | 35 | 308 | 6.558113 | Phagocytosis |
| 5.21E-23 | 56 | 576 | 5.61083 | **Neutrophil degranulation** |
| 5.67E-23 | 56 | 584 | 5.533969 | **Neutrophil activation involved in immune response** |
| 9.56E-23 | 56 | 593 | 5.44998 | **Neutrophil mediated immunity** |
| 1.25E-22 | 56 | 597 | 5.413464 | **Neutrophil activation** |
| 2.46E-22 | 56 | 606 | 5.333066 | Granulocyte activation |
| 9.56E-23 | 58 | 639 | 5.23828 | Leukocyte degranulation |
| 5.67E-23 | 59 | 652 | 5.22235 | **Myeloid cell activation involved in immune response** |
| 4.01E-22 | 58 | 660 | 5.071607 | **Myeloid leukocyte mediated immunity** |
| 7.26E-24 | 66 | 785 | 4.852168 | **Myeloid leukocyte activation** |
| 5.67E-23 | 69 | 903 | 4.409841 | Regulated exocytosis |
| 1.53E-17 | 54 | 717 | 4.346465 | **Blood vessel development** |
| 4.04E-18 | 56 | 748 | 4.320639 | **Vasculature development** |
| 1.55E-20 | 63 | 843 | 4.312951 | **Leukocyte activation involved in immune response** |
| 1.82E-20 | 63 | 847 | 4.292583 | **Cell activation involved in immune response** |
| 4.59E-22 | 72 | 1027 | 4.045979 | Exocytosis |
| 9.66E-17 | 62 | 975 | 3.669853 | Leukocyte mediated immunity |
| 2.01E-27 | 103 | 1658 | 3.585207 | Cell activation |
| 1.70E-20 | 77 | 1245 | 3.569299 | **Immune effector process** |
| 5.67E-23 | 89 | 1475 | 3.482247 | Leukocyte activation |
| 2.65E-17 | 68 | 1127 | 3.482143 | **Circulatory system development** |
| 9.04E-22 | 87 | 1489 | 3.371989 | Secretion by cell |
| 5.67E-23 | 94 | 1639 | 3.309866 | **Cell adhesion** |
| 6.99E-23 | 94 | 1646 | 3.29579 | **Biological adhesion** |
| 2.54E-21 | 88 | 1545 | 3.287121 | Export from cell |
| 7.45E-21 | 90 | 1636 | 3.174832 | Secretion |
| 4.13E-17 | 82 | 1590 | 2.976311 | **Cell migration** |

**Supplementary table 5 :** Enriched pathways (KEGG) associated with SLC16A1 where cancer associated terms are indicated in **Bold fonts**.

| Enrichment FDR | nGenes | Pathway Genes | Fold Enrichment | Pathway |
| --- | --- | --- | --- | --- |
| 1.13E-06 | 13 | 36 | 6.882850242 | **DNA replication** |
| 0.069686 | 4 | 13 | 5.864677129 | Non-homologous end-joining |
| 2.34E-11 | 29 | 108 | 5.118016846 | Nucleocytoplasmic transport |
| 3.02E-11 | 31 | 126 | 4.68941445 | **Cell cycle** |
| 0.006342 | 9 | 41 | 4.183946488 | Homologous recombination |
| 0.106671 | 5 | 23 | 4.143521885 | Mismatch repair |
| 0.032676 | 7 | 33 | 4.043072869 | Base excision repair |
| 5.78E-06 | 20 | 97 | 3.929938282 | MRNA surveillance pathway |
| 0.00298 | 11 | 54 | 3.88263347 | Fanconi anemia pathway |
| 0.014323 | 9 | 46 | 3.729169696 | Nucleotide excision repair |
| 1.16E-06 | 25 | 132 | 3.60988649 | Spliceosome |
| 0.001488 | 14 | 77 | 3.465491031 | Ribosome biogenesis in eukaryotes |
| 0.052132 | 9 | 56 | 3.063246536 | **Hedgehog signaling pathway** |
| 0.000176 | 22 | 141 | 2.973932019 | Ubiquitin mediated proteolysis |
| 1.02E-11 | 70 | 495 | 2.695381913 | Herpes simplex virus 1 infection |
| 0.064137 | 15 | 129 | 2.216302403 | Oocyte meiosis |
| 0.126141 | 16 | 156 | 1.954892376 | Cellular senescence |
| 0.111906 | 21 | 222 | 1.802991955 | Human T-cell leukemia virus 1 infection |

| Enrichment FDR | nGenes | Pathway Genes | Fold Enrichment | Pathway |
| --- | --- | --- | --- | --- |
| 4.48E-17 | 38 | 124 | 5.84102924 | DNA geometric change |
| 1.77E-16 | 40 | 143 | 5.33152466 | Double-strand break repair via homologous recombination |
| 1.95E-19 | 63 | 294 | 4.08432871 | Double-strand break repair |
| 6.06E-35 | 124 | 639 | 3.69869309 | DNA repair |
| 2.32E-18 | 68 | 354 | 3.66128148 | DNA recombination |
| 7.38E-18 | 70 | 381 | 3.50187414 | Nucleocytoplasmic transport |
| 9.75E-18 | 70 | 384 | 3.47451575 | Nuclear transport |
| 1.77E-16 | 66 | 366 | 3.43708537 | DNA conformation change |
| 2.12E-48 | 192 | 1092 | 3.35124407 | Chromosome organization |
| 3.78E-41 | 176 | 1056 | 3.17670011 | **DNA metabolic process** |
| 8.13E-26 | 118 | 725 | 3.10221197 | **Cell cycle phase transition** |
| 1.54E-35 | 163 | 1021 | 3.04291157 | **Mitotic cell cycle process** |
| 5.42E-20 | 97 | 615 | 3.00624303 | **Mitotic cell cycle phase transition** |
| 2.32E-18 | 91 | 585 | 2.9649201 | **Regulation of cell cycle phase transition** |
| 1.72E-32 | 156 | 1005 | 2.95859831 | Cellular response to DNA damage stimulus |
| 5.67E-37 | 179 | 1166 | 2.92605139 | **Mitotic cell cycle** |
| 9.40E-18 | 91 | 599 | 2.89562314 | MRNA processing |
| 2.59E-20 | 106 | 705 | 2.86578904 | **Microtubule cytoskeleton organization** |
| 1.01E-25 | 133 | 889 | 2.85152608 | **Regulation of cell cycle process** |
| 3.98E-42 | 218 | 1496 | 2.77748913 | **Cell cycle process** |
| 1.50E-16 | 94 | 659 | 2.71875396 | **Cell division** |
| 1.67E-44 | 265 | 1997 | 2.52927049 | **Cell cycle** |
| 1.93E-23 | 149 | 1126 | 2.52217575 | RNA processing |
| 7.20E-25 | 159 | 1209 | 2.50667651 | **Regulation of cell cycle** |
| 8.47E-18 | 124 | 978 | 2.41663076 | Microtubule-based process |
| 1.22E-19 | 180 | 1631 | 2.10351693 | Negative regulation of cellular macromolecule biosynthetic process |
| 2.26E-19 | 180 | 1642 | 2.08942516 | Negative regulation of macromolecule biosynthetic process |
| 3.92E-18 | 174 | 1607 | 2.06376784 | Negative regulation of nucleobase-containing compound metabolic process |
| 8.47E-18 | 180 | 1702 | 2.0157674 | Negative regulation of cellular biosynthetic process |
| 9.88E-18 | 182 | 1734 | 2.00055163 | Negative regulation of biosynthetic process |

**Supplementary table 6 :** Enriched pathways (GO-BP) associated with SLC16A1 where cancer associated terms are indicated in **Bold fonts**.
