# Supplementary figures and images for: A systemic analysis of monocarboxylate transporters in ovarian cancer and possible therapeutic interventions
Source: Channels (Austin). 2023 Nov 7;17(1):2273008. doi: 10.1080/19336950.2023.2273008 (PMC10631444; doi:10.1080/19336950.2023.2273008)

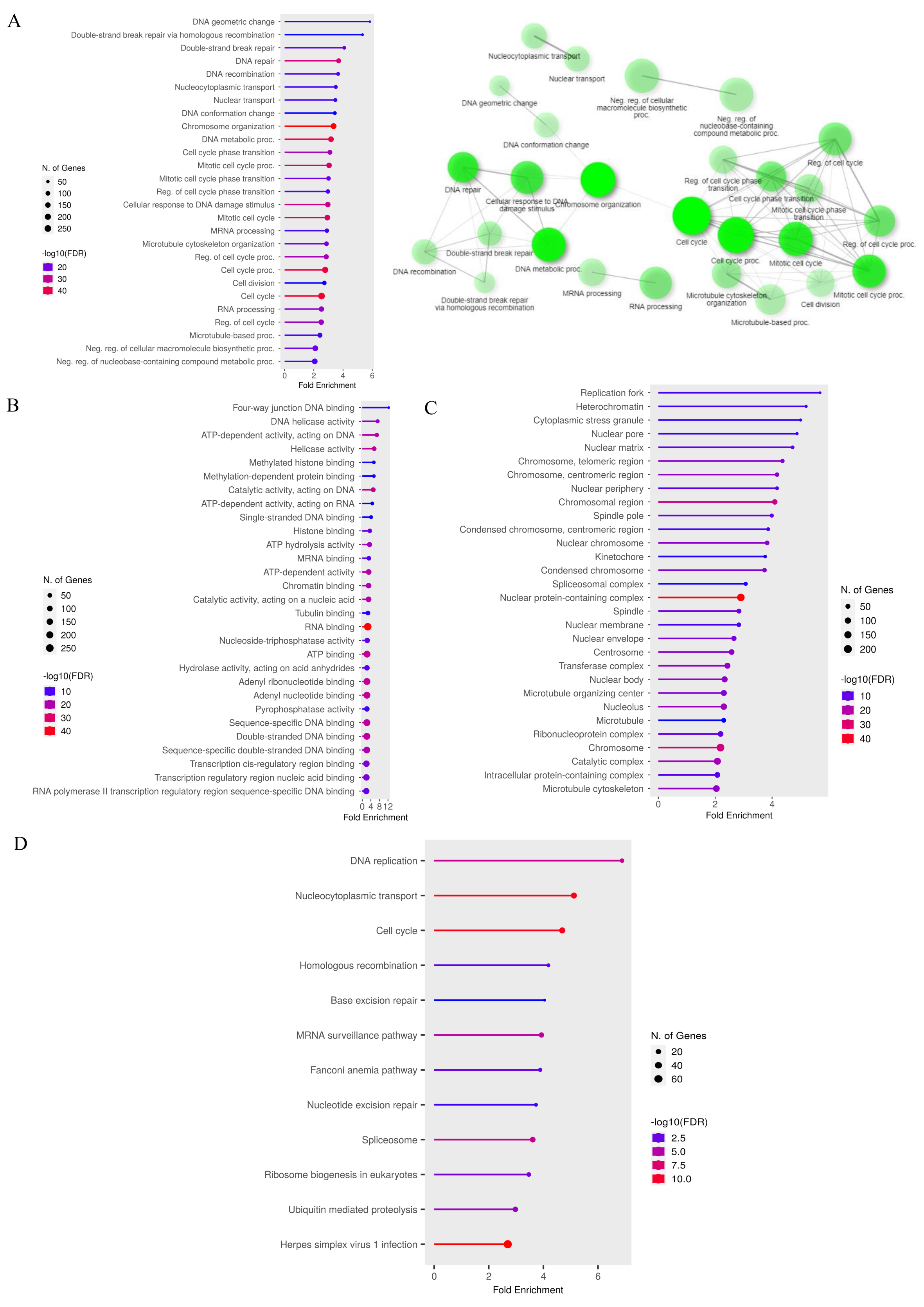

Supplement: Supplemental Material [file KCHL_A_2273008_SM4276.zip › Supplementary files/supplementary_figure_1.tif]

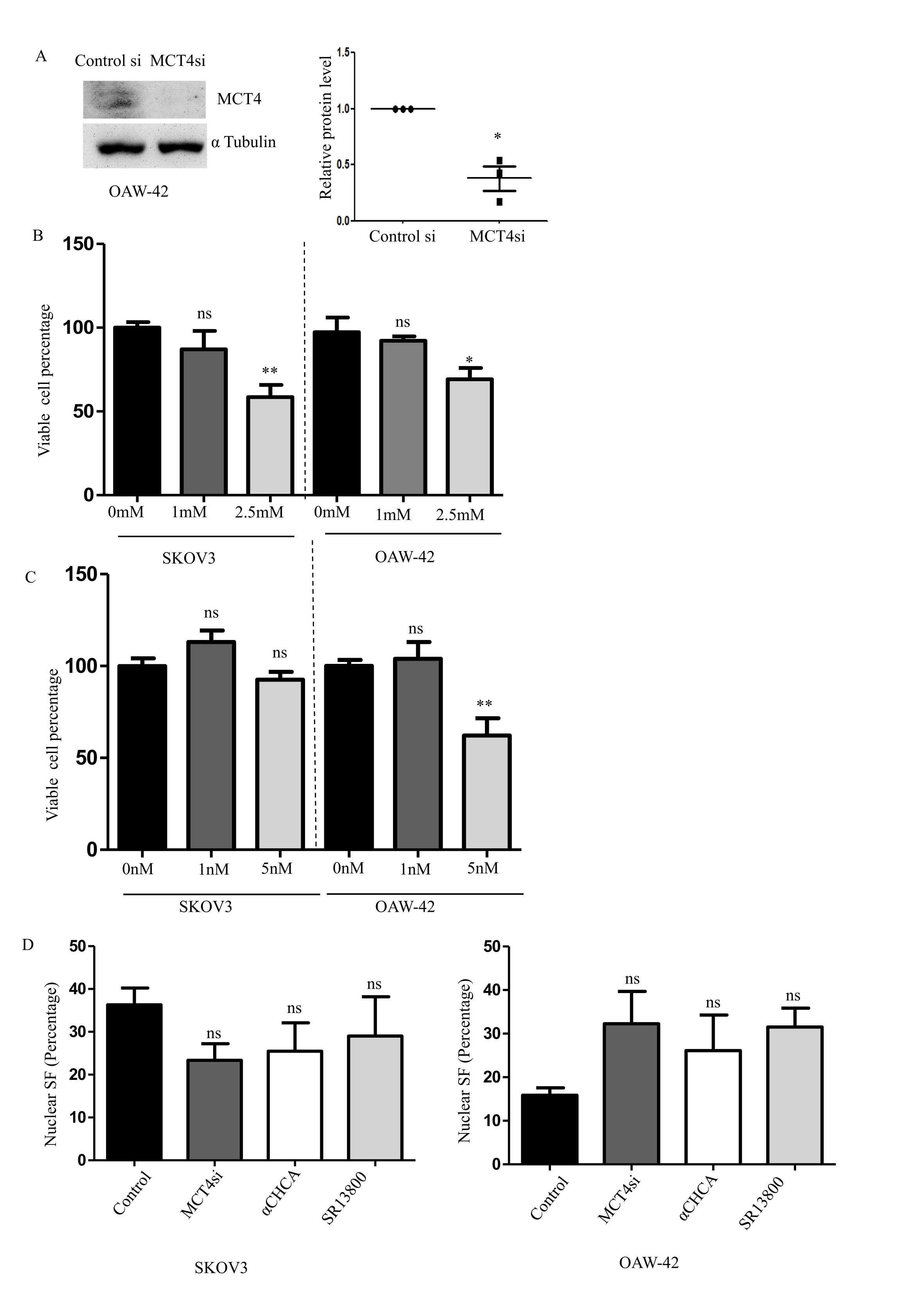

Supplement: Supplemental Material [file KCHL_A_2273008_SM4276.zip › Supplementary files/supplementary_figure_2.tif]

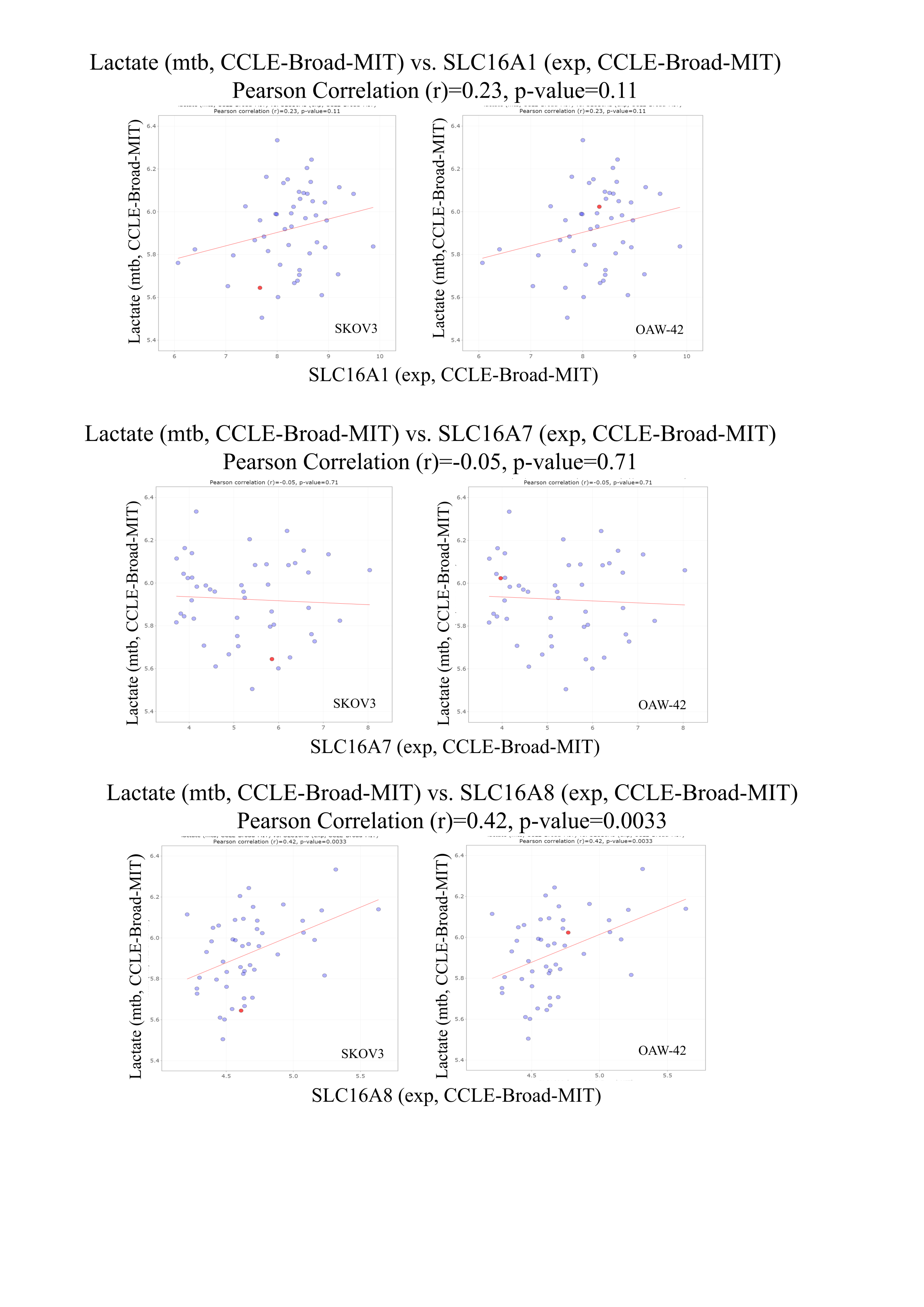

Supplement: Supplemental Material [file KCHL_A_2273008_SM4276.zip › Supplementary files/supplementary_figure_3.tif]
